# Economic Burden of Diabetic Mellitus Among Patients on Follow-up Care in Hospitals of Southwest Shewa Zone, Central Ethiopia

**DOI:** 10.1186/s12913-022-08819-0

**Published:** 2022-11-23

**Authors:** Addisu Bogale Zawudie, Dawit Wolde Daka, Dejene Teshome, Meskerem Seboka Ergiba

**Affiliations:** 1 Ethiopian Public Health Institute, Addis Ababa, Ethiopia; 2 Department of Health Policy and Management, Faculty of Public Health, Jimma University, Jimma, Ethiopia; 3 Pathfinder International Ethiopia, Addis Ababa, Ethiopia

**Keywords:** Direct cost, Indirect cost, Total cost, Intangible cost, Economic burden, Diabetes mellitus, Hospital, Oromia, Ethiopia

## Abstract

**Background:**

Diabetes has emerged as one of the most serious health issues of the twenty-first century. Diabetes and its complications expose individuals and their families to catastrophic healthcare costs, which have a severe impact on the country's economy. Though the prevalence of diabetes is rising quicker in Ethiopia, little is known about its economic impact. Hence, this study aimed to determine the total cost of diabetic mellitus and associated factors among patients attending hospitals in Southwest Shewa zone, Central Ethiopia.

**Methods:**

The study was conducted among diabetes patients who were on care and treatment from September to October 2020. Direct costs were calculated using the micro-costing technique, while indirect costs were calculated using the human capital approach. The statistical significance of cost difference between the groups of patient characteristics was determined using Wilcoxon and Kruskal-Wallis mean rank sum tests, and the factors associated with a total cost of illness were identified with Generalized Linear Model (GLM).

**Results:**

Out of the planned patients, 398 have responded and were included in the analysis; making a response rate of 98.5%. The mean monthly total cost of diabetic mellitus was US$ 37.7(95% CI, 23.45–51.95). Direct and indirect costs constituted 76.2% and 23.8% of the total cost, respectively. The mean direct and indirect cost of diabetic mellitus per patient per month was US$ 28.73(95% CI, 17.17–40.29) and US$ 9.50 (95% CI, 1.99–16.99) respectively. Statistical mean cost differences were observed by gender, age groups, family size, and comorbidities. The total cost of illness was associated with residence (*p=*0.007), family size (*p=*0.001), presence of co-morbidities (*p=*0.04), and history of ever-stopping treatments (*p<*0.0001).

**Conclusions:**

The total cost of diabetes condition was relatively high compared to other related literatures. The medical expenditures accounted for most direct costs for diabetic patients. As a result, the government should provide sufficient resources to safeguard patients against catastrophic medical costs. Efforts should be made to enhance access to diabetes care, and the supply of diabetic medications at all levels of health facilities.

## Introduction

Diabetes mellitus (DM) is a chronic disease that arises when the pancreas produces insufficient insulin or when the body's insulin is not used correctly. There are two major types of diabetes; type 1 and type 2 diabetes. Type 1 diabetes (T1DM) develops in childhood and adolescence and requires lifelong insulin injections for survival. Whereas, type 2 diabetes (T2DM) develops in adulthood and accounts for 90% of diabetic cases worldwide [1].

Diabetes mellitus has emerged as one of the major public health issues of the twenty-first century and it is a leading cause of death, morbidity, and healthcare expenses worldwide [1, 2]. According to the latest estimates from the International Diabetic Federation (IDF), over 425 million people worldwide had diabetes in 2017, and in 2045, this number will climb to 629 million [3, 4]. Diabetes prevalence has increased quicker in low- and-middle-income nations than in high-income countries during the last decade. In 2017, 39 million Africans have diabetes and this figure will rise to an estimated 82 million by the end of 2045 [5].

Globally the cost of diabetes hits 825 billion dollars a year with the largest cost to individual countries being in China ($170 billion), the Unites States ($105 billion), and India ($73 billion) [5]. The global costs of diabetes and its consequences are large and will substantially increase to $2.1 trillion by 2030 [5, 6]. Diabetes cost the US economy at least $727 billion (about $2,200 per person in the US) in 2017, accounting for 12% of total adult consumption [7]. The total estimated cost of diagnosed diabetes in 2017 is $327 billion, including $237 billion in direct medical costs and $90 billion in reduced productivity [8]. Diabetes is a costly disease to manage in low-and-middle-income countries. The outpatient costs per visit ranged from under US$ 5 to over US$ 40 (median: $7), and the annual inpatient costs ranged from approximately US$10 to over US$1000 (median: $290). The annual laboratory costs ranged from under US$5 to over US$100 (median: $25); and annual medication costs ranged from $15 to over $500 (median: $177). Care for complications was generally incur a high-cost, and varied widely across countries and complication types [8]. Diabetes imposes a substantial economic burden in countries of low-and-middle incomes that requires increased investments and intensified action on the prevention and control of the disease [9, 10].

Diabetes is on the high rise in Ethiopia. The prevalence of diabetes in Ethiopia ranged from 2.0% to 6.5% with a low prevalence of 2% in rural areas [11], and the pooled prevalence of diabetes mellitus was 4.99% [12]. Diabetes and related complications are major causes of morbidity and mortality with consequential economic impact. The frequently reported diabetes-associated problems included retinopathy, neuropathy, depression, kidney diseases, hypertension, and anemia [11, 12]. The increased prevalence of diabetes mellitus in the past decades is related to lifestyle changes with significant population growth and urbanization [13–15].

Diabetes and its consequences have a significant financial and economic impact on people, families, and governments [7]. When access to cheap insulin is limited, those with diabetes who rely on it for survival pay the ultimate price [16]. World leaders have pledged to decrease the burden of diabetes as one of four priority NCDs to address this rising health concern. Member states have set an ambitious goal to reduce premature mortality from NCDs, including diabetes, by one-third, achieve universal health coverage, and provide affordable essential medicines, considering global NCD targets for 2025 and NCD-related Sustainable Development Goal targets for 2030 [17].

Ethiopia is one of the United Nations signatories, and it is projected to meet objective 3.4, which states that "by 2030, reduce premature death from non-communicable illnesses by one-third" [18]. Recognizing the rising burden of NCD, the Ministry of Health has prepared a comprehensive preventive and control strategic action plan and risk factors focused on the reduction of hazardous behaviors [19].

In Ethiopia despite the increase prevalence and burden of diabetes, little is known about its economic burden. A study conducted in Northern Ethiopia revealed that the economic burden of diabetes is high with the less privileged groups (less educated, the poorest, and unemployed) being disastrously affected. Sixty percent of diabetes patients incurred catastrophic diabetes care expenditure and 5% of diabetes were impoverished by diabetes care spending US$ 1.9 per day [20]. Similarly, a study conducted in the same region as the earlier article showed that diabetes mellitus is an expensive illness and the direct medical cost constituted a major segment in the treatment of the illness. The total direct and non-medical costs per year were 12, 721.5 USD and out of these direct medical costs constitute the larger share, which is 86.5% [21]. Another study conducted in the capacity city, Addis Ababa, indicated that diabetes mellitus is an expensive illness to manage in individuals with low income. The median monthly direct cost of care for a diabetes patient was $21.8 and the median lost days of patients due to an illnesses was 6 days [22].

The cost of illness is determined by several factors, including the type of disease, the number and severity of complications, the demographic characteristics of the study population, access to medical care, and the quality of care received. The presence of diabetic complications or related comorbidities, diabetes medication, and hospitalization represented the biggest portion of the economic burden for diabetes [20, 21, 23]. Higher socioeconomic class individuals spent more on self-care and other pharmaceuticals, and this difference was significant in both direct and indirect expenses [24, 25]. The cost difference was much larger among customers who had diabetes for a longer time, while the indirect cost difference between the groups was not significant in this study [24].

Measuring the economic impact of diabetes and understanding the major causes of economic burden has paramount importance programmatically. Examining all factors helps to obtain an accurate picture and comprehensive knowledge of economic burden related to diabetes mellitus. The evidence can help policymakers, planners, and program managers to look for strategies that protect citizens from financial hardships related to the diseases. Therefore, this research aimed to determine the economic burden of diabetes among patients who are on care and treatment in public hospitals in Southwest Shewa zone, Oromia region, Central Ethiopia. The study estimates direct and indirect costs of illness and intangible burdens of diabetes illnesses.

## Methods and materials

### Study setting and period

The study was conducted in the hospitals of the Southwest Shewa zone of the Oromia regional state, central Ethiopia from September to October 2020. Southwest Shewa is one of the zonal administrations in the bigger regional state of Ethiopia (that is Oromia) located 114 kilometers in the Southwestern direction of Addis Ababa. The study zone comprised 11 districts, one town administration, 22 urban Kebeles and 264 rural Kebeles. As of 2020, the total estimated population of the zone was 1, 101, 129 with a male population of 556, 194 and a female population of 544, 935. The residents in the urban accounted 14% (149, 878) of the population. In terms of health service coverage, the zone had 5 hospitals, 54 health centers, 264 health posts, and 72 private clinics. Four hospitals are owned by the government and the remaining hospital is governed by a non-profit organization. The hospitals provides diabetes care and treatment services to the catchment population in the zone and people in the neighboring zonal administrations [26].

### Study design and participants’

A cross-sectional study was conducted among diabetes patients who were on a follow-up care and treatment services in the study hospitals. Patients who had less than one year of follow-up, incomplete information on medical records, were unable to listen and speak, and were not willing to participate in the study were excluded. The number of patients included in the study were determined using single population proportion formula (OpenEpi sample size calculator) [27] assuming 50% prevalence (patients who were on follow-up and cost for their health assumed), 95% confidence level, and 5% of margin of error to obtain a maximum sample size. After adding 5% of the non-respondents rate the calculated sample size yielded 404.

The sample size was proportionally allocated to each of the hospital based on the monthly patient load. The monthly patient load of hospitals was retrieved from the District Health Information Software (DHIS2) (a reporting tool). The average number of patients per month who were recorded in the hospital and that meet the criteria of the Ethiopian Revised National Disease Classification (NCOD) ID of 6577 and 6578 was 1,561. The total patients and the allocated sample size of the study hospitals were: St. Luke (*N=*763, *n=*197), Tulo Bolo (*N=*408, *n=*106), Amaya (*N=*151, *n=*39), Bantu (*N=*131, *n=*34), and Leman (*N=*108, *n=*28); where ‘N’ is the total patient per month, and ‘n’ was allocated sample size. On the date of the survey, patients were approached on their exit from services, consent was taken and interviewed. Patients were selected by employing simple random sampling technique using patients records as a sampling frame. Moreover, patient records were reviewed and data recorded on the data abstraction template.

### Measurement

A structured questionnaire was developed based on relevant literatures [28, 29] and modified to match the aim of this study. The questionnaire was divided into four components. The first component describes patient characteristics, and the second focused on the expenses of diabetes mellitus diagnosis and treatment. Component three is related to an intangible cost or burden of illness and the final section comprises items related to knowledge and attitudes of patients towards diabetes and its management. The questionnaire was primarily prepared in English, translated to local language (Afan Oromo) by experienced translator and then back translated to English with an independent translator to maintain its consistency. Following this, pretest was conducted in a non-selected healthcare facility in the adjacent zone and modifications were made on the questionnaire as appropriate.

Four health professionals with a qualification of bachelors of science in nursing, who have sufficient experience working in a chronic care clinic, and proficient in local language were recruited outside of the study area and participated in data collection. Another health professional with a qualification of bachelors of science in public health was recruited and participated in the overall supervision of data collection process. A two-days training was provided to the field data collection team on the questionnaire, data collection techniques and research ethics.

The data collection team approached the study hospitals, and continued an interview with patients after taking an informed consent. Interviews were undertaken in the convenient places that maintain the privacy of respondents. As the same time, the data collectors have reviewed records of patients after obtaining a permission from the facility in charge. Supervisors have monitored the overall data collection process and provided support at the field level. Data completeness and consistency were checked daily, and corrections were made as appropriate.

### Study variables

Direct cost: the opportunity cost of resources used for treating diabetes patient that includes direct medical and direct nonmedical costs.

Direct medical cost: an amount of money paid for registration, laboratory investigation and medication/drugs in the study health facilities during the study period.

Direct nonmedical cost: an amount of money paid for transportation, food, and accommodation for patient and their companion during hospital visits.

Indirect costs: the value of resources lost due to diabetes illness. Indirect costs are costs associated with loss of working time of the patients and its companion or the loss of income related to the illness (due to absenteeism, missing business appointment).

Total cost of diabetes mellitus: the cost incurred by patient because of a diabetes problem, typically including both the direct and the indirect costs associated with diabetes.

Intangible cost or burden: related to the pain and suffering endured by patients, thus causing a decline in their quality of life, social isolation, and impairment of their personal satisfaction.

### Approaches for cost estimation

The cost of illness analysis was undertaken from the societal perspective and a prevalence-based methodology was employed to estimate cost. The main outcome variable of the study was total cost of diabetes care and treatment among patients. The cost of diabetes illness was the cost spent by patients and their family as the consequence of diabetes illness (that is the cost for treatment and care of diabetes). The cost attributed to the illnesses was calculated for a one-month period (that is 30-days) and then converted to a one-year period by multiplying with 12. The total cost of illness was the sum of direct and indirect costs. The determination of direct and indirect expenses was based on a study of patient records and patient memory (interviews).

The direct cost was estimated using a micro-costing or bottom-up approach [30–32]. Direct cost is the opportunity cost of resources that are used for treating diabetes patients. This cost constitutes direct medical and direct non-medical costs. The direct medical costs are costs of outpatient visits, laboratory tests, medicines, and costs related to other services (such as the use of self-monitoring blood glucose and consumables). Whereas the direct non-medical costs comprised of cost for transportation and lodgings. The direct cost data were collected from patient records and interviews. Transport costs were collected from a patient for round trips and the number of visits made to each care provider or hospital. Lodging costs were estimated from patient-reported costs and the number of days of stay in hospitals.

The indirect cost is the productivity loss due to diabetes illness and was estimated in terms of the productive time lost by patients and their companions during travel and consultation with the healthcare provider. The human capital approach was used to value the productivity time lost [33] and the patient and their companions time was valued using opportunity cost method. First, the time forgone in seeking care and productivity time lost were estimated. Then, the time forgone was converted into cost based on the daily wage rate. For those patients who were employed or had regular monthly income the total hours or days of productivity lost was multiplied by the average hourly or daily earnings. The local daily wage rate for unskilled labor was obtained from the patient response. The indirect costs of unemployed patients and those who were not in the labor force were not considered because the opportunity cost of the time according to human capital approach is nil. All direct and indirect cost information was collected in Ethiopian Birr (ETB) and then converted to US$ (US$1=35.205 ETB as of August 2020 exchange rate).

Intangible cost or burden of illness was related to the pain and suffering endured by patients, thus causing a decline in their quality of life, social isolation, and impairment of their personal satisfaction. Intangible burden was measured by 8 items with each having a five point Likert scale that ranged from 1=strongly disagree to 5=strongly agree, and another one items that asks about the perceived quality of life of the patients. The intangible burden items were: (a) I am not able to enjoy my hobbies since the diagnosis of the disease; (b) I have sleepless nights because of diabetes condition; (c) I have low self-confidence because of diabetes condition; (d) I have low self-esteem because of diabetes condition; (e) I avoid company of others because of my diabetes condition; (f) I easily get fatigued because of my diabetes condition; (g) I easily change diet because of diabetes; and (h) I easily get irritated because of my diabetes condition. The question that asks about their quality of life was ‘scale of one to five, how would you rate your quality of your life’. This question was ranged from ‘1=very poor to 5=very good’.

### Data processing and analysis

Data were checked for completeness and consistency, entered to Epidata version 3.1 and then exported to SPSS version 25 for analysis. Descriptive statistics was used to analyze and present the characteristics of patients and various cost items of diabetes. Direct and indirect costs and intangible burden of illnesses were analyzed separately, and the total cost was calculated as the sum of direct and indirect costs. All cost data were presented in US$ using mean, median, and standard deviations.

A composite intangible burden of illness indice was constructed. The scores were categorized as ‘low (9-20), medium (21-32) and high (33-45)’ intangible burden of illness using the descriptive statistics tertile approach [28]. Fishers’ exact test was used to determine associations between intangible burden of illness and patients’ characteristics.

Normal distribution of cost data was assessed using Shapiro-Wilk test and the result revealed that data were not normally distributed (*P<*0.001). As a result, we have used non-parametric tests such as Wilcoxon and Kruskal-Wallis mean rank sum tests to evaluate statistical significance mean cost differences across the patient characteristics. Moreover, Generalized Linear Model (GLM) was used to estimate coefficients and identify the factors associated with the total cost of diabetes condition adjusting for age and sex. GLM permits flexible modeling of covariates and enable inference to be made directly about the mean costs, rather than focusing on transformation methods. It helps to determine the relationship between a dependent variable and a set of predictor variables, and allow response variable to have other than normal distribution [34]. In all analysis *p*-value <0.05 was considered as statistically significant. All analysis was performed using SPSS version 25 (IBM corporation, New York, USA).

## Results

### Characteristics of patients

The diabetes cost analysis was performed using total of 398 diabetic patients with a response rate of 98.5%. The patients’ average age was 51 years (SD 14.74). The minimum and maximum age were 18 and 90 years. The majority of patients were male (56.5%), married (72.9%), orthodox religion followers (50.0%), and with no formal education (42.0%). The main occupation categories of patients were merchant (23.1%), farmer (20.1%), and government employee (19.1%). The average family size in the household of a patient was 6 (SD 2.0) with a minimum and maximum family size of 1 and 9, respectively. The majority of patients (58.3%) were from urban areas and on average the patients have travelled 21.3 kilometers (SD 19.47) to visit hospitals for diabetes treatment and care with a minimum and maximum distance of 2 and 165 kilometers, respectively. On average household of a patient had an income of US$ 175.17 (SD 129.32) per month with minimum income of US$ 28.41 and a maximum income of US$ 890.0. Whereas, average monthly income of a patient per month was US$ 96.85 (SD 86.17) (Table 1).

**Table 1:** Characteristics of diabetes patients in hospitals of Southwest Shoa Zone, Central Ethiopia, 2020

Variables		Frequency	Percent
Observations		398	
Sex	Male	225	56.5
	Female	173	43.5
Age	18-24	16	4.0
	25-34	36	9.0
	35-44	69	17.3
	45-54	88	22.1
	55-64	107	26.9
	Greater than 65	82	20.6
Residence	Urban	232	58.3
	Rural	166	41.7
Marital Status	Single	74	18.6
	Married	290	72.9
	Widowed	26	6.5
	Divorced	8	2.0
Religion	Orthodox	199	50.0
	Muslim	94	23.6
	Protestant	99	24.9
	Catholic	6	1.5
Educational Status	No formal education	167	42.0
	Primary education	68	17.1
	Secondary education	75	18.8
	Higher education	88	22.1
Main Occupation	Unemployed	101	25.4
	Employed (payroll paid)	125	31.4
	Farmer	80	20.1
	Merchant	92	23.1
Family Size	1-3	43	10.8
	4-6	209	52.5
	Greater than 6	146	36.7
Distance from hospital in kilometers	Less than 10	158	39.7
	11-20	69	17.3
	21-30	83	20.9
	31-40	42	10.6
	41-50	19	4.8
	Greater than 50	27	6.8
Household monthly income (Mean± SD)		398	117.6 (±68.7)
Patients’ monthly income (Mean± SD)		398	98.6 (±186.17)

*Abbreviations: SD* Standard deviation

The average duration of diabetes illness was 6 years (SD 5.72). Majority of the patients had an illness duration that ranged from one to five years (64.6%) followed by 6-10 years (26.4%). Greater than seven out of ten (73.6%) patients had co-morbidity and diabetes complications. On average patients had 3 complications (SD 1.44). The major complications were hypertension (91.8%), dyslipidemia (41.0%), depression (30.7%), retinopathy (38.2%), and neuropathy (34.5%) (Table 2).

**Table 2:** Clinical characteristics of diabetes patients

Clinical characteristics	Frequency	Percent
Observations	398	
Duration of diabetes illness in years		
1-5	257	64.6
6-10	105	26.4
11-15	17	4.3
>15	19	4.8
Presence of co-morbidity and complications		
Yes	293	73.6
No	105	26.4
Complications^a^		
Hypertension	269	91.8
Dyslipidemia (high cholesterol)	120	41.0
Retinopathy (eye damage)	112	38.2
Neuropathy (nerve disease)	101	34.5
Nephropathy (kidney damage)	47	16.0
Foot Ulcer	6	2.0
Skin conditions	24	8.2
Hearing impairment	60	20.5
Alzheimer's disease (brain disorders)	4	1.4
Depression	122	41.6

^*a*^*Out of patients that had complications (*n=*293)*

Metformin and Nifedipine (17.4%); Spironolactone Tab and Hydrochlorothiazide (HCT) (13.2%); Glimepiride and Enalapril (9.3%); and Insulin and Nifedipine (8.9%) were the most often utilized medications for the management of diabetes complications among the patients in the study area (Table 3).

**Table 3:** Drugs used to manage diabetes complications

Drugs	Frequency	Percent
Observations	281	
Metformin + Nifedipine	49	17.4
Spironolactone Tab + Hydrochlorothiazide (HCT)	37	13.2
Glimepiride + Enalapril	26	9.3
Insulin + Nifedipine	25	8.9
Spironolactone Tab + HCT + Nifedipine	16	5.7
Simvastatin + HCT	15	5.3
Clindamycin Caps+ Lisinopril	12	4.3
Insulin + Hydrochlorothiazide (HCT)	12	4.3
Amoxicillin Clavulanic acid + Nifedipine	9	3.2
Miconazole + Nifedipine + Enalapril	9	3.2
Insulin + HCT + Nifedipine	7	2.5
Lisinopril + Hydro Tab + Atenolol + Nifedipine	7	2.5
Clindamycin Caps + Nefedipine + Enalapril	6	2.1
Clotrimazole + Nifedipine + Hydrochlorothiazide (HCT)	6	2.1
Others^a^	45	16.0

^*a*^*Acetylsalicylic Acid Tab + Enalapril + HC (n=4), Erythromycin Tabs + Enalapril (n=4), Insulin + Enalapril (n=4), Simvastatin + HCT + Nifedipine (n=4), Simvastatin + Paracetamol (n=4), Fluconazole + Enalapril + nifedipine (n=3), Glimepiride + Nifedipine (n=3), Insulin, Nifedipine + Enalapril (n=3), Metformin + Nefedipine + Lasix (n=3), Metformin + Nefedipine + Enalapril + paracetamol (n=3), Simvastatin + Enalapril (n=3), Metformin + Enalapril (n=2), Metformin + Nifedipine + Enalapril (n=2), Amoxicillin Clavulanic acid + Nifedipine+ Enalapril (n=1), Glimepiride + Nifedipine + Lasix (n=1), Simvastatin + Nifedipine + Enalapril (n=1)*

### Costs of diabetes mellitus

#### Direct cost

The average monthly direct cost of diabetes illness was US$ 28.73 (SD 11.60) [95% CI: 27.59, 29.87] and the mean annual direct cost was US$ 344.77 (SD 139.17) [95% CI: 331.10, 358.44]. The mean monthly and annual direct medical costs were US$ 17.80 (SD 7.06) [95% CI: 17.11, 18.49] and US$ 213.62 (SD 84.74) [95% CI: 205.30, 221.95]. Similarly, the mean monthly and annual direct non-medical costs were US$ 10.96 (SD 7.83) [95% CI: 10.19, 11.73] and US$ 131.49 (SD 93.94) [95% CI: 122.26, 140.72], respectively. Overall, the direct monthly medical expenses of patients were US$11,435.03 and out of which the direct medical cost constituted 61.96%.

Overall patients have spent a total of US$ 2,784.92 for insulin (Mean± SD: US$7.00±3.28), US$ 469.54 for insulin syringes (Mean± SD: US$1.20±0.69) and $4519.56 for medicines of diabetes complications (Mean± SD: US$1.85±2.02). The average cost of laboratory tests was US$ 1.43 with a cost that ranged from US$ 0.57 to US$ 9.09.

Diabetic mellitus patients' total non-medical cost was US$4,350.04. On average patients have spent US$4.43 for food, US$1.47 for transportation and US$ 1.93 for physiotherapy per a month period. The majority of patients (256 or 64.3%) have utilized buses to go to the hospital, and a quarter of them (96 or 24.1%) were utilized motorbikes or Bajaj as a means of transportation. While the remaining (46 or 11.6%) have used other mechanisms of transportation (including horses and on foot) (Table 4).

**Table 4:** Direct costs for diabetes mellitus care and treatment (Exchange rate: US$1 = ETB 35.205)

Cost items	Observations	Sum	Mean	Median	Std. Deviation
Total Direct cost of DM	398	$11,435.03	$28.73	$26.99	11.56
Direct medical cost	398	$7,085.00	$17.80	$16.30	7.06
Cost of registration/ folder	398	$334.61	$0.84	$0.57	0.54
Cost of consultation fees	280	$255.28	$0.91	$0.28	1.30
Cost of self-monitoring of glucose	300	$655.42	$2.18	$1.79	1.86
Cost of Insulin	398	$2,784.92	$7.00	$6.25	3.28
Cost of Insulin syringe/ disposable	392	$469.54	$1.20	$1.08	0.69
Cost of oral anti-hyperglycemic agents (OHA)	213	$464.79	$2.18	$2.56	0.89
Cost of drugs used for complications	281	$519.56	$1.85	$1.28	2.02
Cost of laboratories	391	$557.51	$1.43	$0.57	1.38
Cost of non-prescribed remedies	283	$1,043.37	$3.69	$2.84	4.94
Direct non-medical cost	397	$4,350.04	$10.96	$9.66	7.83
Cost of physiotherapy	132	$254.88	$1.93	$1.56	1.81
Cost of food and drink	380	$1,681.81	$4.43	$3.41	4.56
Cost of dressing	241	$1,376.79	$5.71	$5.68	3.80
Cost of transportation	394	$736.17	$1.87	$1.14	2.01
Cost spent by companion for diabetes care	80	$300.38	$3.75	$2.84	4.39

#### Indirect costs

During a one-month recall period, diabetes patients and their caregivers have spent time seeking care from the hospital and physiotherapists. Out of 398 diabetic patients that visited the hospital, 302 (86.5%) had family or friends with them, while 47 (13.5%) have come alone. One hundred fifty-six (51.6%) of the companions had lost part of their income because of their care.

In a one-month period, 388 patients have spent 470 working days seeking care from the hospital, an average of one day per patient. While 394 patients have lost 252 hours (15, 111 minutes) because of visiting hospitals in the specified period. Moreover, a total of 373 days were spent by 294 companions with a median of 1 day. Patients and their companion had spent a total of 843 days with a median of 2 days per month that should be used for productive activities.

The total indirect expense for diabetes care and treatment in the one-month recall period was US$ 3561.93. The total loss of earnings (indirect cost) for patients was US$2,144.01 with an average of US$ 5.91 (SD 4.35). This cost constituted the expenses of patients (loss of earnings) because of visiting hospitals for care and treatment services of diabetes. The companion's average loss of earnings due to diabetes was US$6.20 (SD 2.27) (Table 5).

**Table 5:** Indirect costs for diabetes care and treatment (Exchange rate: US$1 = ETB 35.205)

Indirect costs	Observations	Sum	Mean	Median	Std. Deviation
Patients income lost (US$)	363	$2,144.01	$5.91	$5.11	4.35
Patients absent from work (in days)	388	470	1.21	1.00	0.90
Patients travel time to the hospital (in minute)	394	15,111	38.35	30.00	32.75
Accompany income lost (US$)	232	$1,417.92	$6.11	$5.68	6.39
Accompany absent from work (in days)	294	373	1.27	1. 00	0.92
Accompany travel time to hospital (in minute)	302	10,899	36.09	20.00	33.54
Patients and accompany absent from work (in days)	394	843.00	2.14	2.00	1.35

#### Total costs

The total monthly diabetes management cost of patients was US$ 14,996.97 with the total direct cost accounting of 76.2 percent of the total cost. The direct medical costs accounted of 62% of the total direct cost and 47.2% of the total cost. The cost of medicines had a high contribution to the direct medical costs. The direct medical costs were twice as much as the indirect costs (Table 6).

**Table 6:** Total cost of diabetes

Diabetes costs	Observations	Sum	Mean	Median	Std. Deviation
Total direct cost	398	$11,435.03	$28.73	$26.99	11.56
Direct medical cost	398	$7,084.99	$17.80	$16.30	7.06
Direct non-medical cost	397	$4,350.04	$10.96	$9.66	7.83
Total indirect cost	375	$3561.94	$9.50	$7.67	7.50
Total cost	398	$14996.97	$37.68	$36.50	14.25

#### Intangible burden of diabetes

Four out of ten patients were unable to enjoy their hobbies due to diabetes illness (40%) and had a low self-confidence because of their diabetes condition (40.5%). The majority of patients got fatigued (71.4%), changed diets (82.4%), and were easily irritated (72.6%) because of their diabetes condition. Less than half (47.2%) of patients perceived that their quality of life was Good (Table 7).

**Table 7:** Intangible burden diabetes among patients

Intangible burden items	Frequency	Percent
Observations	398	
I am not able to enjoy my hobbies since the diagnosis of the disease		
Agreed	159	40.0
Disagreed	239	60.0
I have sleepless nights because of diabetes condition		
Agreed	228	57.3
Disagreed	170	42.7
I have low self-confidence because of diabetes condition		
Agreed	161	40.5
Disagreed	237	59.5
I have low self-esteem because of diabetes condition		
Agreed	177	44.5
Disagreed	221	55.5
I avoid company of others because of my diabetes condition		
Agreed	200	50.3
Disagreed	198	49.7
I easily gets fatigued because of my diabetes condition		
Agreed	284	71.4
Disagreed	114	28.6
I easily change diets because of my diabetes condition		
Agreed	328	82.4
Disagreed	70	17.6
I easily get irritated because of my diabetes condition		
Agreed	289	72.6
Disagreed	109	27.4
Perceived quality of life		
Good	188	47.2
Poor	210	52.8

The majority of patients (284 or 71.4%, 95% CI: 66.8, 75.6) had a medium intangible burden of diabetes illness, and one-fifth (82 or 20.6%, 95% CI: 16.8, 24.8) had a high intangible burden associated with diabetes illness. While the remaining one-tenth (32 or 8%, 95% CI: 5.7, 11.0) patients had a low intangible burden of illness. A statistical significant intangible burden of diabetes illness difference was observed only in the education category of patients and no difference was observed in the remained characteristics of patients (Table 8).

**Table 8:** Intangible burden of diabetes illness by patient characteristics

Variables	Intangible costs			*P*-value
	Low (n,%)	Moderate (n,%)	High (n,%)	
Observations	32	284	82	
Sex				
Male	15 (46.9)	168 (59.2)	42 (51.2)	0.23
Female	17 (53.1)	116 (40.8)	40 (48.8)	
Age				
18-24	2 (6.3)	10 (3.5)	4 (4.9)	0.46
25-34	0 (0.0)	25 (8.8)	11 (13.4)	
35-44	6 (18.8)	51 (17.9)	12 (4.2)	
45-54	10 (31.3)	64 (22.5)	14 (4.9)	
55-64	8 (25.0)	74 (26.1)	25 (8.8)	
>=65	6 (18.8)	60 (21.1)	16 (5.6)	
Educational status				
No formal education	15 (46.9)	129 (45.4)	23 (28.0)	0.04*
Primary education	5 (15.6)	40 (14.1)	23 (28.0)	
Secondary education	6 (18.8)	50 (17.6)	19 (23.2)	
Higher education	6 (18.8)	65 (22.9)	17 (20.7)	
Residence				
Urban	17 (53.1)	165 (58.1)	50 (61.0)	0.74
Rural	15 (46.9)	119 (41.9)	32 (39.0)	
Duration of illness in years				
1-5	19 (59.4)	184 (64.8)	54 (65.9)	0.77
6-10	12 (37.5)	71 (25.0)	20 (24.4)	
11-15	0 (0.0)	14 (4.9)	3 (3.7)	
>15	1 (3.1)	15 (5.3)	5 (6.1)	
Presence of co-morbidity				
Yes	20 (62.5)	212 (74.6)	59 (72.0)	0.33
No	12 (37.5)	72 (25.4)	23 (28.0)	

*P*-value: Chi-squared and Fisher Exact tests value, significant difference at *P<*0.05

### Association of patients’ characteristics and diabetes cost

Male patients had a higher indirect cost (US$ 122.07, *p=*0.001) compared to female patients. A statistical significance direct cost and total cost difference was observed in relation the presence of co-morbidity and family size. Patients with a co-morbidity had a higher direct (US$ 210.11, *p=*0.002) and total cost (US$ 212.95, *p<*0.0001) of diabetes illness compared with those with no co-morbidity. Similarly, patients with a family size of greater than six had a higher direct (US$ 219.81, *p=*0.01), and total cost (US$ 215.64, *p=*0.005) of diabetes illness compared to their counterparts. While, patients in the age group of 55-64 (US$ 223.49, *p=*0.01) had a higher direct cost of diabetes illness (Table 9).

**Table 9:** Association of diabetes costs with patients’ characteristics

Characteristics	Direct Cost		Indirect Cost		Total cost	
	Mean Rank (US$)	*P*-Value	Mean Rank (US$)	*P*-Value	Mean Rank (US$)	*P*-Value
Sex^a^						
Male	200.39	0.86	122.07	0.001*	207.45	0.12
Female	198.34		94.40		189.16	
Residence^a^						
Urban	205.96	0.19	113.81	0.34	206.02	0.18
Rural	190.47		105.34		190.38	
Presence of co-morbidity^a^						
Yes	210.11	0.002*	109.60	0.65	212.95	*P<*0.0001*
No	170.64		114.69		162.91	
Family size^b^						
1-3	166.14	0.01*	92.50	0.19	150.23	0.005*
4-6	192.17		116.62		198.36	
Greater than 6	219.81		105.10		215.64	
Age category^b^						
18-24	149.88	0.01*	65.83	0.08	144.94	0.15
25-34	171.26		134.26		184.14	
35-44	185.70		98.97		185.68	
45-54	217.09		104.41		210.55	
55-64	223.49		104.28		215.45	
≥65	183.02		128.03		195.84	
Level of education^b^						
No formal education	211.25	0.18	103.85	0.44	207.74	0.38
Primary	200.85		112.28		205.24	
Secondary	176.32		124.26		180.88	
Higher	195.92		112.69		195.30	
Duration of illness^b^						
1-5	196.05	0.62	109.56	0.75	195.75	0.59
6-10	203.07		111.67		200.89	
11-15	193.18		132.63		209.76	
>15	229.33		103.07		230.21	

* Significant level at p<*0.05*

^*a*^*Wilcoxon Rank Sum test was used to determine the significant difference in costs distribution*

^*b*^*Kruskal-Wallis test was used to determine the statistically significant differences in costs distribution*

### Drivers of total cost of illness

In the Generalized Linear Model analysis, four characteristics of patients (residence, family size, presence of co-morbidity and ever stopped diabetes treatment) had shown statistically significant associations with the total cost of diabetes illness after adjusting for age and sex. Educational status has showed a marginal associations and the rest of the variables didn’t shown associations. The total cost of diabetes illness was four times higher in urban patients as compared to the rural one (Coefficient estimate=4.45, 95% CI: 1.20,7.69, *p=*0.008), and the total cost increases by 26% with a unit increase in the size of family (Coefficient estimate=1.26, 95% CI: 0.54,1.98, *p=*0.001). Moreover, the total cost of diabetes illness was three times higher among patients with co-morbidities (Coefficient estimate=3.17, 95% CI: 0.05,6.28, *p=*0.04), and six times higher in patients who ever stopped diabetes treatments as compared with their counterparts (Coefficient estimate=6.17, 95% CI: 3.11,9.22, *p<*0.0001) (Table 10).

**Table 10:** Factors associated with total cost of illness

Variables	Frequency (%) or Mean *± SD*	Coefficient Estimate (95% CI)	*P*-value
Observations	398		
Age in years (Mean *± SD)*	51.1 (14.74)	-0.08(-0.24,0.08)	0.34
Sex			
Male	225 (56.5)	-5.08(-15.23,5.07)	0.33
Female	173 (43.5)	Ref	
Educational status			
No formal education	167 (42.0)	Ref	
Primary education	68 (17.1)	-2.21(-6.28,1.87)	0.29
Secondary education	75 (18.8)	-4.01(-8.38,0.37)	0.07
Higher education	88 (22.1)	-4.39(-9.14,0.36)	0.07
Residence			
Urban	232 (58.3)	4.45 (1.20,7.69)	0.007*
Rural	166 (41.7)	Ref	
Marital status			
Married	290 (72.9)	1.86(-1.36,5.07)	0.26
Others	108 (27.1)	Ref	
Family size (Mean *± SD)*	5.9 (1.99)	1.26 (0.54,1.98)	0.001*
Main occupation			
Unemployed	101 (25.4)	Ref	
Employed	125 (31.4)	2.69(-1.52,6.90)	0.21
Farmer	80 (20.1)	1.67(-3.18,6.53)	0.50
Merchant	92 (23.1)	0.13(-4.07,4.33)	0.95
Distance from the hospital in kilometers (Mean *± SD)*	21.3 (*19.47)*	-0.008(-0.08,0.07)	0.83
Duration of illness in years (Mean *± SD)*	5.7 (5.51)	0.13(-0.12,0.38)	0.31
Presence of co-morbidity			
Yes	293 (73.6)	3.17 (0.05,6.28)	0.04*
No	105 (26.4)	Ref	
Ever stopped diabetes treatment			
Yes	107 (26.9)	6.17 (3.11,9.22)	*p<*0.0001*
No	291 (73.1)	Ref	

*Abbreviations:* *SD* Standard deviation, *CI* Confidence interval

## Discussion

The study revealed that the total cost of diabetes condition among patients per month was US$ 14996.97 with a mean cost of US$ 37.68. The larger portion of the total cost (76.2%) was constituted by the direct costs including direct medical and non-medical costs, and the direct medical cost has shared 47.2% of the total costs. The patients were exposed to intangible burden of illness with one-fifth of patients reported to have a high intangible burden of illness (20.6%) and the majority had a moderate burden of illness (71.4%). A statistically significant mean cost difference was observed by patients gender, age groups, family size, and presence of co-morbidity; while a statistical difference in intangible burden of illness shown only in patients’ education category. Residence, family size, presence of co-morbidity and ever-stop treatment were associated with the total cost of diabetes illness.

The cost of diabetes condition per month was higher in the study area compared to findings from India [35, 36], lower than a study conducted in Ghana [28, 37], and much lower than studies done in the USA [38], China [39], South Korea [23], Indonesia [40], Nigeria [41] and Kenya [42]. The cost of diabetes conditions in the study setting was also higher as compared to studies done in Northern Ethiopia [21] and in Addis Ababa health facilities [22]. The variations of diabetes care and treatment cost implied the differences in the assessment period and contextual factors including the nation’s economy, health system, and patient characteristics.

The direct cost of diabetes care and treatment has the larger share of the total cost and it is twice as high as the indirect costs. The direct medical cost including the cost of a folder, consultation fees, drugs, and laboratory tests accounted for nearly half of the total cost of diabetes care in patients. This is comparable to findings from other parts of the world [10, 23, 28] and in Ethiopia [21, 22]. The cost of insulin including disposable syringes and the cost of non-prescribed remedies constituted six out of ten (60.2%) of the direct medical costs. A drug cost has shared a high portion of the total cost as evident from a study done in Africa countries [9]. Whereas, the cost of laboratory tests accounted for nearly one-tenth of diabetes care and the cost of drugs for diabetes complications management constituted 7.3% of the direct medical costs. The direct non-medical costs have also a significant contribution to the total cost of diabetes care and treatment. The direct non-medical cost that included the costs for physiotherapy, food and drinks, dressings, and transportation has contributed to 38% of the total direct cost. This has a huge implication for the patients and society at large exposing them to catastrophic healthcare expenditure.

The patients and their companion had spent a total of 843 days with a median of 2 days per month that should be used for productive activities. In terms of costs, they have spent US$ 3561.93 (mean cost $9.50) with an average cost of US$ 5.91 for patients and US$6.20 for their companions owing to missed incomes. This finding is higher than that of a study done in Ghana [28] and lower than that of Addis Ababa health facilities [22]. However, the result showed that diabetes has a detrimental influence on working productivity with the indirect cost (loss of earnings) expenses accounting for nearly a quarter (23.8%) of the overall cost of care and treatment. This negative influence on production has been observed in numerous nations with a variety of social and economic settings [6, 38].

A statistical mean cost difference was observed whenever taking a look at the characteristics of patients such as gender, age groups, family size, and presence of comorbidity. Male patients had a higher indirect cost (US$ 122.07, *P=*0.01) and total costs (US$207.45, *P=*0.016) compared to female patients. Patients with a co-morbidity had a higher total cost of diabetes illness compared with those without co-morbidity and patients within a family size of greater than six had a higher direct, indirect, and total cost of diabetes illness compared to their counterparts. This is comparable to a finding from other parts of the world [21, 22, 39, 42].

Previous studies reported that majority of patients with diabetes are more likely to suffer various degree of intangible burden ranging from moderate to high related to physical pain, psychological pain, social relationship, functional limitation and quality of life. The management of diabetes disease using insulin injection for a longer period is maybe inconvenient and psychologically stressful [43–45]. In the present study majority of patients (71.4%) had a moderate intangible burden of illness, and one-fifth had a high intangible burden of illness. There was a statistically significant relationship between intangible burden of illness and the education status of patients, while in the remaining patients’ characteristics there were no associations. This contradicts to a study done in Ghana where no statistical relationship was observed in all of the socio-demographic characteristics [28]. Intangible illness burden negatively influences outcome of diabetes management and this requires regular monitoring [43].

In the study area, residence, family size, presence of co-morbidities and ever stoppage of diabetes treatments were associated with the total costs of illness. Patients from urban areas, larger family size, with co-morbidities and ever stopped treatments had a higher cost of illness. The total cost of illness was four times higher in urban patients compared with rural patients (*p=*0.007). Other study also revealed that urban patients spent more on diabetic care than rural patients [46]. The mean total cost of illness among urban patients was US$ 38.6 with a total cost of US$ 8945.2 and that of rural patients was US$ 36.5 with total cost of US$6051.8. The majority of patients in urban areas had co-morbidities (60.5% vs 39.5%) and experienced ever stoppage of diabetes treatments (60.7% vs 39.3%) as compared to rural areas. This might cause the increase in cost of illnesses. Previous studies has also reported that diabetes cost of illness is higher in patients with complications [28, 47, 48]. Besides, patients located in the urban settings were easily accessible to care and treatment centers and were economically stable as compared to those in the rural areas [49–51]. Thus, they might more likely spent on diabetes care and treatments.

The present study also revealed that total cost of illness is higher in patients who had co-morbidities and a history of drug or treatment stoppage. Patients who had co-morbidities spent three times a higher cost of illness (*p=*0.04) and those patients who ever stopped treatments spent six times higher cost of illness compared to their counterparts (*p<*0.0001). This is partly related to complications arising due to failure to take medications and this in turn will resulted to an additional cost for diabetes complication management [52]. In our study out of the total patients who experienced drug stoppage, most (88.8%) had complications such as hypertension, dyslipidemia, retinopathy, neuropathy, and nephropathy in the study area. Complications had high impact on the cost of diabetes care and treatments [53, 54].

The study was conducted in a larger sample size, and have estimated the direct and indirect costs and intangible burden of diabetes conditions. But, it is not apart from limitations in that; (a) intangible costs were not evaluated in monetary terms and the productivity losses owing to presentism and costs of early deaths were not considered in the indirect cost estimations; (b) the time analysis was limited and focused only one month of care; and (b) patients are recruited at the hospital level and this might impose a bias in the sample selection. Regardless of these drawbacks, however, the findings of the study can be used to inform evidence-based policymaking and program planning related to diabetes illness.

### Policy implications

Similar to reports from other settings of the world [52], the cost of diabetes illness is higher in the study area. With an increasing rise of diabetes prevalence, this might expose patients to a catastrophic healthcare expenditure and causes burden on the country’s economy. This has huge impact on a country with a low economy. One of the challenge related to diabetes is its complications in that most of the complications requires an advanced management centers [55]. Diabetes patients living in low-income settings face unique challenges related to lack of awareness, difficulty in accessing healthcare systems and medications, and consequently failure in achieving the optimal diabetes management and prevention of complications [56]. It is reported that in LMICs fewer than one in ten people with diabetes receive guideline-based comprehensive diabetes treatment [57]. Therefore, this requires much effort from the government in strengthening diabetes prevention and control, and addressing the challenges of access to care and treatments. Effective diabetes prevention and care models including standardization of prevention and management guidelines, improving access to care, engaging community and peers, improving training of professionals, and use of newest technology in the management of the diseases could help to reduce the rising burden of diabetes.

## Conclusions

The study concluded that the total cost of diabetes illness was higher among patients in the study area. This has huge implications for individual patients and society at large. The majority of diabetes care and treatment expenditure is borne by direct costs with about half of the total cost accounted for direct medical costs. Equally importantly the indirect costs including the cost of productivity losses due to the illness had a significant contribution to the total cost of diabetes illness. Similarly, the study also showed that most of the patients (92%) mitigated intangible burden of illness with moderate intangible illness burden accounting for 71.4% and that of the high intangible burden accounting for 20.6%. The cost of diabetes condition was associated with residence, family size, presence of co-morbidities and history of ever-stopping treatments. As a result, effort should be made to improve access to diabetes care and treatment so that patients can obtain the required services in nearby healthcare facilities. Moreover, diabetes prevention and control interventions should be strengthened at all levels of the health system with a particular focus to the primary healthcare level. We also suggest a look at the policy to consider cost subsidization strategies particularly for the medical costs and if possible availing insulin and other relevant medication free for the poorest group of the population.

## Data Availability

The datasets used and/or analyzed during the current study are not openly available because data is part of ongoing study or research project and available from the corresponding author (dave86520@gmail.com or dawit.daka@ju.edu.et) on reasonable request. The data will be accessible openly when the research is completed.
